# Biologically Enhanced Patch in the Healing and Mechanical Stability of Rotator Cuff Tears

**DOI:** 10.26502/jbb.2642-91280161

**Published:** 2024-09-04

**Authors:** Merlin Rajesh Lal LP, Devendra K Agrawal

**Affiliations:** Department of Translational Research, College of the Osteopathic Medicine of the Pacific, Western University of Health Sciences, Pomona, California 91766, USA

**Keywords:** Biological patch, Rotator cuff, Tendon injury, Tissue engineering

## Abstract

Biological patches have emerged as promising adjuncts in the surgical management of rotator cuff tears, aiming to enhance tissue healing and biomechanical properties of repaired tendons. These patches, derived from human or animal sources such as dermis or small intestinal submucosa, undergo mechanical and pathological changes within the rotator cuff environment post-implantation. These patches provide structural reinforcement to the repair site, distributing forces more evenly across the tendon and promoting a gradual load transfer during the healing process. This redistribution of forces helps alleviate tension on the repaired tendon and surrounding tissues, potentially reducing the risk of re-tears and improving overall repair integrity. Moreover, biological patches serve as scaffolds for cellular infiltration and tissue ingrowth, facilitating the recruitment of cells and promoting collagen synthesis. The integration of these patches into the host tissue involves a cascade of cellular events, including inflammation, angiogenesis, and matrix remodeling. Inflammatory responses triggered by patch implantation contribute to the recruitment of immune cells and the release of cytokines and growth factors, fostering a microenvironment conducive to tissue repair. However, despite their potential benefits, the long-term efficacy and durability of biological patches in rotator cuff repair remain areas of ongoing research and debate. Further studies are needed to elucidate the optimal patch characteristics, surgical techniques, and rehabilitation protocols to maximize clinical outcomes and minimize complications in rotator cuff surgery.

## Introduction

Rotator cuff tear is a prevalent and debilitating musculoskeletal condition, inflicting considerable impairment on affected individuals [1]. With an estimated incidence of 17% in the general population over 50 years old [2], rotator cuff tears significantly impact quality of life, functional capacity, and occupational performance. These tears commonly result from acute trauma, chronic overuse, or age-related degeneration, leading to disruptions in the integrity of the rotator cuff tendons [3, 4]. As the rotator cuff plays a pivotal role in stabilizing the glenohumeral joint and facilitating shoulder movements, tears can manifest as pain, weakness, and limited range of motion [4]. The underlying pathogenesis and clinical symptoms are primarily due to inflammation, disorganization of extracellular matrix, activation of inflammasomes, fatty infiltration, and local effect of immunological factors [5–9]. Several co-morbidities, such as hyperlipidemia, diabetes, and smoking, and the in vivo hypoxic environment worsen the pathophysiology [10–14]. Some of the tendons may undergo chronic adaptation upon injury [15]. However, several potential targets have been identified and exosomes and hydrogel network have been used in treatment strategies [16–19].

While conservative management options such as physical therapy and corticosteroid injections may suffice for partial tears or mild symptoms, surgical intervention often becomes necessary for full-thickness tears or failed conservative treatments [20,21]. Surgical repair aims to re-establish tendon continuity, restore shoulder biomechanics, and alleviate symptoms, thereby improving functional outcomes and patient satisfaction [4]. Various surgical techniques, including arthroscopic and open approaches, are employed based on tear size and tissue quality [22,23]. Despite advancements in surgical techniques and rehabilitation protocols, rotator cuff repair remains challenging with a failure rate of 20–90% with outcomes influenced by factors such as tear size, tendon quality, patient age, and underlying co-morbid conditions [24,25]. The ongoing research efforts focusing on optimizing surgical strategies [23,26,27] and rehabilitation protocols [28–30] have been discussed elsewhere, while enhancing tendon healing biology to improve outcomes has been discussed in this article.

## Biological Patch

Various biological patches are available for tissue repair, each utilizing different tissue sources and processing methods to enhance regeneration. A list of patches available for rotator cuff repair are mentioned in Table 1. ArthroFlex Patch, derived from human dermis, undergoes meticulous processing to remove cellular elements while preserving the extracellular matrix (ECM) structure, essential for tissue regeneration [32]. Similarly, the ReGenTec Patch, sourced from porcine small intestinal submucosa (SIS), is processed to maintain the ECM architecture, aiding tissue repair [39]. The GraftJacket patch, derived from human dermis, undergoes decellularization to eliminate cells while retaining the ECM structure, promoting tissue regeneration [34]. Additionally, patches like the Restore Patch, derived from human or porcine dermis, are processed to eliminate cellular and immunogenic components, leaving a biocompatible collagen matrix for effective tissue repair [33]. These diverse biological patches offer clinicians a range of options to cater to specific patient needs, ensuring successful outcomes in tissue regeneration and repair. Biologically enhanced patches represent a cutting-edge approach in tissue engineering and regenerative medicine, offering a promising solution for tissue repair and regeneration [40]. These patches are meticulously engineered to incorporate bioactive materials that closely mimic the native tissue microenvironment, thereby facilitating cellular adhesion, proliferation, and differentiation [40,41]. Among the key components integrated into these patches include extracellular matrix (ECM) scaffolds, growth factors, and cells, each playing a crucial role in enhancing tissue healing and regeneration [42,43]. Acellular human dermal tissue and synthetic biocompatible polymers are increasingly used in clinical practice to strengthen extensive rotator cuff repair (RCR). These materials mainly aim to provide structural and biomechanical support by facilitating load distribution between the scaffold and native tendon tissue or improve biological activity.

### Synthetic Polymer Patches

Traditional polyester grafts have historically aimed to reinforce rather than influence underlying tendon healing. Recent endeavors have explored novel materials, biological enhancements, and nano-scaffold development, showing encouraging short-term results. Scaffolds can also be employed as interposition grafts, either at the enthesis or to bridge massive rotator cuff tears. Larger animal models are beneficial for these studies due to their size, which allows for scaffold applications like human clinical use and repair constructs that mimic those used in patients. Nanofiber scaffold composed of polyglycolic acid and poly-L-lactide-co-E-caprolactone in a sheep model, using a double-row anchor technique for acute infraspinatus tendon repair. They found that interposition scaffolds placed between the rotator cuff footprint and native tendon improved biomechanical properties and some histologic characteristics resembling a native enthesis [44]. The load sharing theoretically helps protect the tendon-to-bone interface and promotes healing. Additionally, these scaffolds can bridge gaps in severely retracted tendons or support fragile native tissue. Scaffolds designed to enhance biological activity differ in composition from those intended for structural support, focusing on promoting cell migration, attachment, proliferation, and extracellular matrix deposition. Currently, numerous scaffolds are used clinically and are considered safe and effective for treating and augmenting large to massive rotator cuff tears [45]. Additionally, rabbit models demonstrated the potential of interposition PGA scaffolds for repair augmentation [46]. Although challenging in rodent models, Cong et al. utilized an electrospun polycaprolactone scaffold in a bridging manner for massive rotator cuff tears, noting enhanced biomechanical and histologic properties [47]. Given the current commercial availability of various scaffold devices in the United States, ranging from polymer-based to human dermis to xenogeneic collagen scaffolds, there are ample research opportunities to refine scaffold composition, placement, fixation methods, and augmentation strategies in future studies.

### Extracellular Matrix Scaffolds

**Figure 1:**
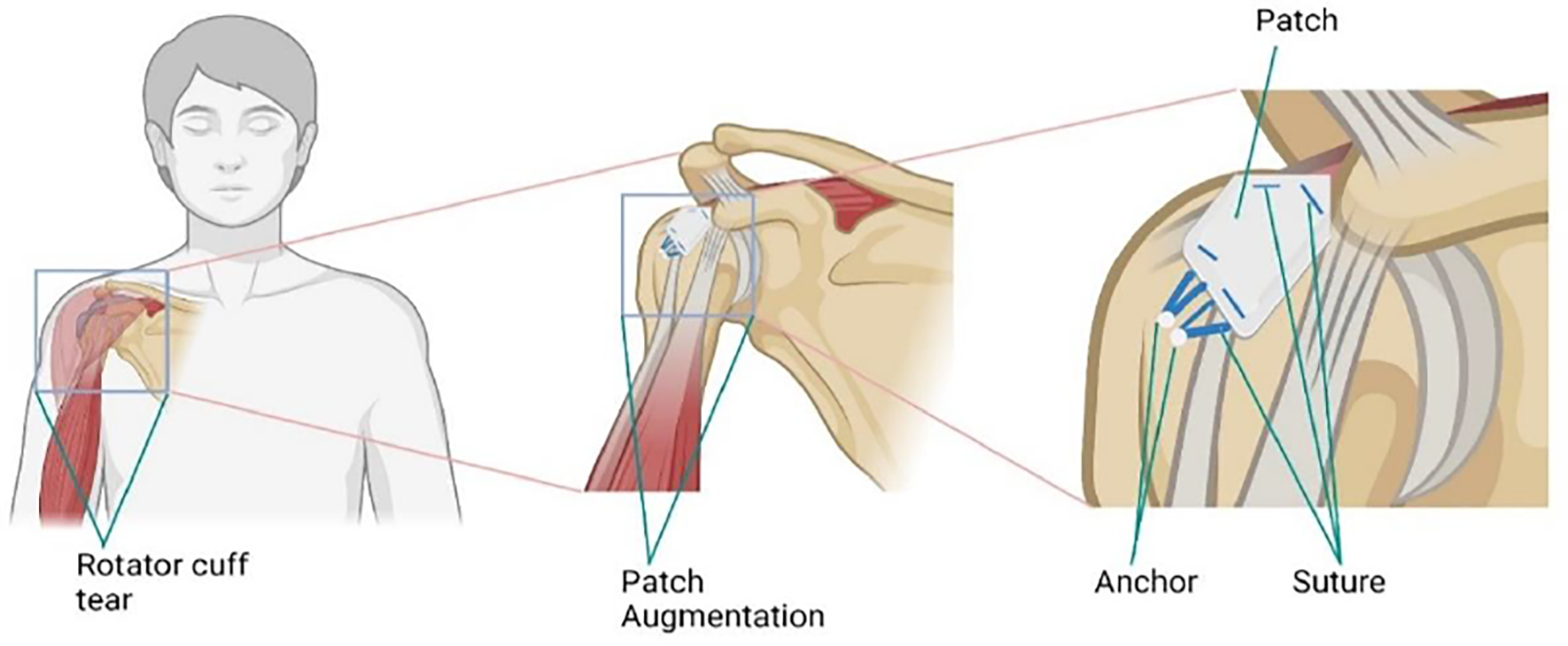
Schematic diagram showing patch augmentation of rotator cuff tendon.

ECM scaffolds serve as a fundamental component of biologically enhanced patches, providing a three-dimensional framework that supports cell infiltration and tissue regeneration [48]. These scaffolds can be derived from natural sources such as decellularized tissues or synthesized using biocompatible polymers. Decellularized ECM retains the native architecture and composition of the tissue, offering an ideal substrate for cell attachment and tissue regeneration [49]. Synthetic polymers, on the other hand, provide greater control over scaffold properties such as pore size, mechanical strength, and degradation kinetics, allowing for customization based on specific tissue engineering applications [50]. The existing research indicates varying degrees of success with patch augmentation, contingent upon the specific graft employed. A schematic representation of rotator cuff repair with graft augmentation is shown in Figure 1. Discrepancies exist among studies utilizing the same material, and the body of evidence is insufficient to endorse any specific graft type. Predominantly, xenografts like porcine dermal or small intestinal submucosa (SIS) grafts are used, although recent findings suggest promising outcomes with human dermal matrix. Porcine dermal grafts have demonstrated positive outcomes in animal studies and may emerge as a dependable option for cuff repair augmentation. Conversely, despite optimistic initial results from animal studies, unsatisfactory outcomes have been observed in humans with porcine SIS grafts [33]. The reason behind this would have been due to the presence of porcine DNA and cellular material in the patch material [51]. Another study which compared the conventional repair with porcine SIS among 62 patients with moderate to large rotator cuff did not show any significant difference in pain, repair failure or patient-reported outcome [52]. Augmentation of supraspinatus tendon was attempted with either autologous fascia lata (FL) or decellularized porcine SIS on rabbit model. The results showed an increase in ultimate load to failure at 12 weeks but had no significance between the porcine SIS and autologous fascia lata. The suture retention on decellularized porcine SIS was reported to be 48.6 ± 5.8 N for single suture and 17.9 ± 2.7 N for double suture [53]. Even though animal studies are reported the histological reports of the use of biological patches in human are very limited or not available on most studies [54]. In another study bovine bio-inductive patch (Rotation Medical, Plymouth, MN) developed from decellularized bovine tendon was used in rotator cuff augmentation of 16 Patients. The external rotation, abduction was increased with no pain or clinical failure after a 3-yeasr follow-up. It was reported that all the patients returned to preoperative sports activities [55,56]. Nevertheless, there is a lack of histological studies comparing these graft types.

Human dermal allograft has exhibited satisfactory outcomes in histological studies, showcasing robust cellular infiltration, revascularization, and new tendon formation [34]. Autografts have also displayed promising histological results in animal models, fostering improved tendon-tendon healing. In rabbit model, freshly harvested autologous periosteum was used to augment rabbit hallucis longus tendon increased the structural integrity of the tendons, but the use on rotator cuff is to be explored. Histological results show a better healing and integration of the graft [56]. In another study, 22 patients underwent arthroscopic or mini-open rotator cuff repair using the patch. Only 41% of patients reached substantial clinical benefit and only 32% of patients reached or exceeded the patient-acceptable symptomatic state (PASS) criteria [57]. These materials are still in early stages and await large-scale clinical trials. Additionally, implantation techniques have been shown to influence healing, with certain centers experimenting with a combination of bone marrow stimulation and patch augmentation to encourage biological healing with positive outcomes. Alongside considerations of potential ineffectiveness, adverse tissue reactions must be acknowledged for all graft types, albeit they are generally rare. In a study on 15 patients who received 16 received porcine SIS, to treat large to massive rotator cuff tendon tare, 4 patient had severe inflammatory reactions to porcine SIS, xenograft which restricts the use graft [58]. A recent clinical study on 7 patient who either received Graft jacket (n=3) or Permacol (n-4) for rotator cuff augmentation, the disruption of native supraspinatus tendon underlying the graft was observed than the control group which did not received conventional suture without patch. Moreover, Histology and IHC analysis after 4 weeks of surgery reported that there was no increase in cellularity or vascularity in both the group compared to the control. Further the Permacol group had infiltration of IRF5+, CD68+, and CD206+ cells, indicating a pro-inflammatory response [37]. But still the comparative study on other grafts are not available to compare. In canine model, different scaffolds, amnion matrix cord scaffold, decellularized human dermal allograft, or bovine collagen patch were used for partial thickness supraspinatus tears. The results showed that decellularized human dermal allografts had the least abnormal MRI pathology scores and histopathology [46]. Earlier it was reported that a 48-year-old patient who underwent rotator cuff augmentation with bio-inductive collagen (Regeneten bio-inductive implant (Smith & Nephew, Andover, MA, USA) and at 4 month developed large swelling with pain but without chills or fever. The patient had no infection, but magnetic resonance imaging (MRI) showed rice bodies like debris in the subacromial-subdeltoid bursa region and a healed rotator cuff tendon. This was thought to be caused by the staples made of polyether ether ketone used to keep the graft in place, which do not dissolve [39]. But it was argued that the dissolution and resorption of these staples would take 12 months and not within 4 months. Even though the rice body formation is reported while using poly-L-lactic acid as orthopedic implants, it is otherwise considered inert like stainless steel and Ethibond suture (Ethicon, Somerville, NJ, USA) [59,60]. Even though the inflammatory reactions and rice body formation was reported with unknown etiology [61] such instances need to be studied in detail before approval for treatment. It is understood that one form of biocompatible polymer need not fit all the requirements. Moreover, modern animal ECM patches undergo more comprehensive DNA extraction procedures, resulting in a diminished inflammatory response, which, for the most part, does not seem to impact clinical outcomes in patients. One notable example of scaffolds in clinical use is bioinductive bovine collagen patches. Small prospective case series have shown their short-term safety [62–65]. Although limited clinical evidence suggests these patches might improve rotator cuff thickness, definitive conclusions are challenging without randomized controlled trials (RCTs) [63].

### Biologically Enhanced Patch

Biologically enhanced patches represent a cutting-edge approach in tissue engineering and regenerative medicine, offering a promising solution for tissue repair and regeneration [40]. These patches are meticulously engineered to incorporate bioactive materials that closely mimic the native tissue microenvironment, thereby facilitating cellular adhesion, proliferation, and differentiation [40,41]. Among the key components integrated into these patches include extracellular matrix (ECM) scaffolds, growth factors, and cells, each playing a crucial role in enhancing tissue healing and regeneration [42,43]. In addition to ECM scaffolds, biologically enhanced patches incorporate a repertoire of growth factors that orchestrate various aspects of the regenerative process [66]. Growth factors, such as platelet-derived growth factor (PDGF), transforming growth factor-beta (TGF-β), and vascular endothelial growth factor (VEGF), exert potent effects on cell behavior, modulating processes such as angiogenesis, matrix synthesis, and inflammation [67]. By incorporating these growth factors into the patch design, researchers aim to create a bioactive microenvironment that promotes tissue regeneration and accelerates the healing process [45]. Furthermore, cell seeded constructs offer a promising avenue for enhancing the regenerative capacity of biologically enhanced patches [68]. These include the transplantation of autologous or allogeneic cells, such as mesenchymal stem cells (MSCs) or progenitor cells, directly into the patch or surrounding tissue [69]. By introducing these cells into the regenerative milieu, researchers aim to replenish damaged cell populations, stimulate endogenous repair mechanisms, and enhance tissue regeneration [70]. MSCs possess unique immunomodulatory and regenerative properties, making them an attractive candidate for cell-based therapies in tissue engineering applications [71–75].

The synergistic combination of ECM scaffolds, growth factors, and cell within biologically enhanced patches holds immense potential for addressing a wide range of clinical challenges [76]. These patches have also been investigated for various applications, including wound healing, bone regeneration, cartilage repair, organ transplantation and tendon regeneration [77]. Advancements in biomaterials science and tissue engineering techniques have led to the development of increasingly sophisticated patch designs with enhanced therapeutic efficacy and clinical translatability [78]. One approach to enhance tendon healing involves using various materials that serve as scaffolds/patches/grafts with platelet-rich plasma, progenitor cells, cytokines and small peptides which in turn support cell migration, attachment, proliferation, and extracellular matrix synthesis [79]. For instance, Arnoczky et al. obtained biopsy samples from patients who had undergone previous RCR augmentation with a porous collagen implant, revealing cellular incorporation, new collagen formation, and resorption of the original implant six months postoperatively [80]. Preclinical models provide more rigorous insights into scaffold activity in vivo. Collagen scaffold was used in a rat rotator cuff model and marked improvement in histologic repair site appearance at 12 weeks were observed but no significant biomechanical enhancements [81].

### Cell-Based Strategies

Augmenting rotator cuff repair (RCR) with cell-based methods has focused on mesenchymal stromal cells (MSCs) or connective tissue progenitors (CTPs) from bone marrow or adipose tissue. In rat models decellularized pericardial membrane seeded with allogenic mesenchymal stem cells ware used for repair of supraspinatus tendon. After three months the supraspinatus tendon of rats that were treated with decellularized pericardium had significantly higher maximum load but no difference in the modulus [36]. These results highlight the early developed tendons [82–84]. Similarly, using a sheep model that received either collagen scaffold or collagen scaffold seeded with autologous tenocytes for rotator cuff repair. The sheep that received collagen scaffold seeded with autologous tenocytes had up to 84% tensile of the native tendons which is significantly higher than the sheep that received collagen scaffolds alone. The histological outcomes were also promising. [85]. Although collagen scaffolds show potential for biological induction, further research is needed to determine if scaffold alone is sufficient or if combined biological strategies are optimal for rotator cuff augmentation. However, inconsistent definitions and low MSC concentrations limit the clinical evidence for these strategies [86]. Better healing of rotator cuff repair with bone marrow aspirate concentrate (BMAC) were observed with 100% healing at 6 months versus 67% in the control group, and 87% intact repairs at 10 years versus 44% in the control group [87]. Similarly, lower failure rates with BMAC augmentation in a randomized controlled trial were reported [88]. Despite promising results, detailed characterization remains insufficient, necessitating further research to correlate clinical outcomes with the composition and activity of the cells.

## Conclusion

In conclusion, biologically enhanced patches represent a paradigm shift in the field of rotator cuff tendon repair, offering a versatile platform for tissue regeneration. By harnessing the synergistic effects of ECM scaffolds, growth factors, and cell-based therapies, these patches provide a conducive microenvironment for tissue regeneration, accelerating the healing process and improving clinical outcomes, but detailed studies are warranted on each modality.

## Figures and Tables

**Table 1:** List of patches used for rotator cuff repair

Sl.no	Product name	Matrix biomaterial	Source	References
1	ArthroFlex allograft	Acellular Dermal Matrix	Human	[31,32]
2	Restore Orthobiologic Implant; DePuy, Warsaw, IN, USA	Small intestine submucosa	Porcine	[33]
3	GraftJacket (Wright Medical, Memphis, TN, USA)	Acellular dermal matrix	Human	[34]
4	SportMesh® (Biomet Sports Medicine, Warsaw, IN)	Knitted fabric device made from Artelon, a resorbable polyurethane urea polymer	Synthetic polymer	[35]
5	OrthADAPT™ (Synovis Orthopedic and Wound Care, Irvine, CA)	Decellularized pericardium	Equine	[36]
6	Permacol(R) or Collagen Repair Patch, (Zimmer Biomet, Warsaw, IN, USA)	chemically crosslinked, acellular dermis	Porcine	[37]
7	GRAFTJACKET NOW	Acellular dermis	Human	[38]
8	Regeneten bio-inductive implant (Smith & Nephew, Andover, MA, USA)	Decellularized tendon	Bovine	[39]

## Data Availability

Not applicable since the information is gathered from published articles.

## Key Points

- Tear of rotator cuff tendons is prevalent among 17% of population over 50 years old and there is a high rate of retear.
- Various surgical techniques are employed to repair rotator cuff tendons depending on the severity of tear followed by appropriate rehabilitation protocol.
- Patch augmentation for repair of tendon has been existing for long term with varying results hence new avenues of treatments are explored.
- Extracellular matrix-based biological patches have been promising but comparable clinical studies with histological data are very limited.
- Biologically enhanced patches with cells or other factors show very good tendon repair in preclinical models but detailed studies need to be conducted for its safety before proceeding to clinical treatment.
